# Inflammatory biomarkers of ischemic stroke

**DOI:** 10.1007/s11739-023-03201-2

**Published:** 2023-02-06

**Authors:** Amedeo Tirandi, Cosimo Sgura, Federico Carbone, Fabrizio Montecucco, Luca Liberale

**Affiliations:** 1 Department of Internal Medicine, University of Genoa, Viale Benedetto XV, 6, 16132 Genoa, Italy; 2 IRCCS Ospedale Policlinico San Martino Genoa, Italian Cardiovascular Network, Largo Rosanna Benzi 10, 16132 Genoa, Italy

**Keywords:** Inflammation, Stroke, Adipocytokines, Adhesion molecules, Cytokines

## Abstract

Ischemic stroke remains the second leading cause of death and among the major causes of morbidity worldwide. Therapeutic options are currently limited to early reperfusion strategies, while pharmacological neuroprotective strategies despite showing promising results in the experimental setting constantly failed to enter the clinical arena. Inflammation plays an important role in the pathophysiology of ischemic stroke and mediators of inflammation have been longtime investigated as possible prognostic marker and therapeutic target for stroke patients. Here, we summarized available evidence on the role of cytokines, soluble adhesion molecules and adipokines in the pathophysiology, prognosis and therapy of ischemic stroke.

## Introduction

Ischemic stroke remains the second leading cause of death and among the major causes of morbidity worldwide. Therapeutic options are currently limited to early reperfusion strategies, which despite the important beneficial effects are burdened by several limitations including the so-called ischemia/reperfusion injury. Indeed, the successful reperfusion increases oxygen and white blood cell availability in the ischemic zone and allow for the increase of inflammatory mediators causing further neuronal death. Given its pivotal role in the pathophysiology of ischemic stroke, inflammation have been long time investigated as a biomarker of stroke outcome and potential target. Yet, none of the anti-inflammatory neuroprotective approaches have successfully passed the bench to bedside transition. Here we summarized available evidence on the role of critical inflammation mediators such as cytokines, adhesion molecules and adipokines in the pathophysiology, prognosis and therapy of ischemic stroke.

## Cytokines

Cytokines are redundant signaling proteins secreted by several cell types involved in stroke pathophysiology, such as endothelial cells, oligodendrocytes, pericytes, astrocytes and immune cells. Cytokines exhibit fundamental growth, differentiation, trafficking and activation functions determining the faith of immune responses and controlling cellular trafficking.

### Tumor necrosis factor alpha (TNF-α)

TNF-α together with IL-1β and IL-6 is usually referred as a primary pro- inflammatory cytokines. In the brain, microglial cells start the local production of TNF-α immediately after the ischemic injury, the migration of circulating inflammatory cells into the infarcted area then favors TNF-α overproduction. TNF-α exerts different effects in the context of ischemic stroke showing both neuroprotective and detrimental characteristics. While TNF-α mediates physiological functions in the brain, being important for the role of glutamatergic neurons and behavioral and cognitive networks [1], TNF-α also associates with BBB impairment, increased inflammatory tension, reactive oxygen species production, and adhesion molecules expression. TNF-α upregulates the expression of metalloproteinases with indirect (via BBB impairment or extracellular matrix remodeling) and direct neurotoxic effects [2]. In accordance with its potential dual role, TNF-α inhibition in experimental models of ischemic stroke showed both beneficial and detrimental effects on stroke size and neurological deficit [3]. Specifically, timing of intervention as well as specific animal model characteristics (such as age, inflammation levels and permanent vs. transient ischemia) have been associated with different outcomes [2, 4]. While TNF-α inhibition may be beneficial on stroke size and neuromotor deficit at short-term ischemia/reperfusion injury, such a protective effect seems lost when looking at long-term outcome, such as post-stroke neuronal plasticity.

TNF-α inhibition during ischemic stroke did not find clinical application, yet. Clinically approved biologicals inhibiting this molecule have been associated with increased incidence of stroke which is thought to derive at least in part from their effect on platelet activation [5]. Instead, TNF-α may hold interesting prognostic roles in such disease. Even though some studies reported no important association between TNF-α levels and the risk of stroke, other studies found that early TNF-α plasma levels correlate with stroke outcome [6]. Also, the concentration of the soluble TNF-α receptor which is higher in the acute phase of stroke can predict the recurrence of vascular events and the occurrence of early seizures in such patients [7]. Indeed, TNF-α was shown to correlate with the presence of small artery lesions. Demonstrating a causal role of this molecule in ischemic stroke pathophysiology in humans, specific TNF-α polymorphisms have been associated with higher risk of ischemic stroke [8].

### Interleukin-1 (IL-1)

Both microglia and infiltrated macrophages produce high levels of IL-1 in the brain parenchyma after an ischemic stroke. IL-1 is associated with BBB impairment and neutrophil infiltration in the brain parenchyma. Mice lacking IL-1α/β have a reduced infarct size after induction of ischemic stroke [9]. Accordingly, administration of IL-1 receptor antagonist (IL-1Ra) favorites a reduction of infarct size in animal model of permanent middle cerebral artery occlusion [10]. Furthermore, specifically blocking IL-1α or -β with monoclonal antibodies already available in the clinic arena at time of reperfusion after an ischemic insult has distinct beneficial effects. While IL-1β antibody can reduce neutrophil infiltration and matrix metalloproteinase-2 activity [11], IL-1α antibody blunts the activation of endothelial cells and the expression of adhesion molecules in the BBB thereby reducing penumbral macrophage content and neurotoxic mediators, such as MMPs [12].

Clinical studies show that higher plasma levels of IL-1β are associated with worst impairment in patients with ischemic stroke [13]. Proinflammatory cytokines increase locally (e.g. in the cerebro-spinal fluid) and systemically after stroke, such increase seems due to the spread of locally produced mediators through the impaired BBB rather than their release by activated circulating cells [13]. Specific polymorphisms of IL-1 receptor antagonist gene are associated with higher risk of ischemic stroke. However, as showed in recent meta-analyses, more studies are needed to draw conclusions about such association [14]. IL-1 pathway may then be a target for reducing the excess of inflammation in stroke patients. IL-1Ra was tested in ischemic stroke in a phase 2 randomized controlled trial in patients presenting within 6 h of acute stroke onset (85% of ischemic and 15% hemorrhagic) [15]. The drug was intravenously given (100 mg bolus at presentation and then 2 mg/kg for 3 days) meeting the tolerability and safety endpoints. Exploratory secondary analysis also gave positive signal towards functional outcome after stroke, with circulating neutrophils, CRP and IL-6 being suppressed in the treatment arm as compared to the placebo one [15]. Intravenous formulation of IL-1Ra were discontinued and another randomized clinical trials evaluated the efficacy of such drug when subcutaneously administered. The SCIL-STROKE (Subcutaneous Interleukin-1 Receptor Antagonist in Ischemic Stroke)—a single-center, randomized, double blind, controlled, phase II study—confirmed tolerability, safety and the effect of IL-1 antagonism on inflammatory mediators upon stroke [16]. The effect on functional outcome was explored by hypothesis-generating mediation analyses showing that subcutaneous IL-1Ra positively affect outcome by reducing plasma IL-6, yet there was a residual negative effect that may suggest interactions with fibrinolysis [16]. Yet, robust phase III clinical trials exploring the effect of IL-1 inhibition on ischemic stroke are still missing to date.

### Interleukin-6

IL-6 is a cytokine with numerous and sometimes opposite effects due to its complex signaling pathways. Indeed, depending on the binding to its receptor which can be found in the membrane-bound or in the soluble form, IL-6 can activate different downstream pathways with regulatory effects on cell growth, differentiation and survival, inflammation and oxidative stress [17]. Within the brain, this molecule is associated with acute inflammation and possible detrimental effects, but also with successful aging [18]. Indeed, the role of IL-6 in course of ischemic stroke is still under scientific debate. Actually, while some studies found that this molecule might not have a direct influence on ischemic stroke, on the other hand, recent evidence shows that IL-6 can exert neuroprotective effects in course of stroke, especially at later time points by facilitating neurogenesis and functional recovery [19, 20].

Clinically, circulating IL-6 is increased after stroke, especially in patients with infarct sizes greater than 3 cm. Furthermore, this molecule showed prognostic abilities. Indeed, IL-6 plasma levels predict stroke size, functional outcome as well as recurrence [21].

### Interleukin-10

IL-10 is an anti-inflammatory cytokine mainly produced in monocyte cells. In the brain, this molecule promotes the survival of both neuronal and glial cells antagonizing the activity of pro-apoptotic cytokines. IL-10 has neuroprotective effect as reported in animal models of stroke with overexpression of IL-10. As a result, these animals have reduced sizes of infarction and neurological deficits. Furthermore, the administration of IL-10 can reduce the post-stroke inflammation in animal models [22].

Clinical studies showed that, low levels of IL-10 are associated with a higher risk of hemorrhagic transformation or neurological deteriorations, whereas high levels with an increased risk of stroke-associated infections [23]. Furthermore, plasma levels of IL-10 are associated with the infarct size [24].

### Adipocytokines

Excess of body weight is known as one of the major risk factors associated with the development of cardiovascular diseases and diabetes. People with high BMI have also an increased risk of stroke although the influence of this finding on prognosis is debated [25]. Several authors have described how increased body weight may have a protective role toward mortality and on outcomes after stroke, in what is called the “obesity paradox”. For this reason, the investigation of adipose tissue and the molecules it produces, may offer interesting research insights [26].

**Fig. 1:**
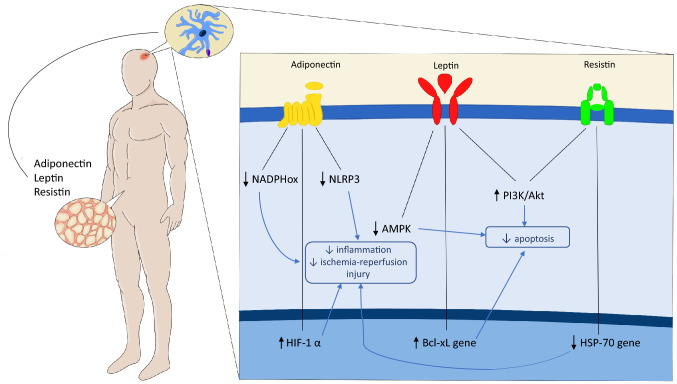
The role of adipokines in ischemic stroke. Although deemed as a mere storage tissue for longtime, nowadays adipose tissue is known to produce several mediators with both local and systemic effects. Such mediators, known as adipocytokines or adipokines are able to modulate inflammatory cell function with different effects on the pathophysiology of ischemic stroke. *AMPK* 5´ adenosine monophosphate-activated protein kinase, *HIF-1α* Hypoxia-inducible factor 1α, *HSP-70* heat-hock protein 70, *NADPHox* nicotinamide adenine dinucleotide phosphate oxidase, *NLRP-3* NLR family pyrin domain containing 3, *PI3K* phosphoinositide 3-kinases

Adipose tissue is involved in the production of several bioactive molecules, those that are mainly but non only produced by adipose tissue are called adipocytokines, those produced solely by adipose tissue are called adipokines (Fig. 1).

### Adiponectin

Adiponectin is a 30 kDa protein firstly isolated in 1995 that shares sequences with collagen-like proteins and a globular domain similar to complement factor C1q.

Adiponectin is produced by healthy adipose tissue and its plasma levels are reduced in states of obesity and insulin resistance due to hypoxia conditions in the hypertrophic and poorly perfused adipose tissue. This protein exerts an anti-inflammatory role on macrophages, reduces the expression of TNF-α and increases insulin sensitivity via binding to AdipoR1 and AdipoR2 receptors. Given the important role that inflammation plays in post-ischemic damage to brain parenchyma, several studies have been conducted correlating adiponectin levels with brain damage in mice. Overexpression of adiponectin improves neurobehavioral outcomes after ischemia in both young and aged mice, even with greater magnitude in the latter group [27]. This neuroprotective effect can be explained by several mechanisms: adiponectin alleviates oxidative damage by inhibiting NADPH oxidase; it reduces ischemia–reperfusion injury by increasing HIF-1α levels and reducing NLRP3 inflammasome activation by regulating AMPK/GSK-3β; furthermore, adiponectin can blunt ischemic injury by phosphorylation of endothelial NO synthase and NO production [28].

Although studies in murine models seems to agree about the neuroprotective role of adiponectin in stroke, the findings from studies in humans are conflicting. In line with the experimental study, early investigations found an association between low levels of adiponectin and increased mortality in patients with stroke [29]. Further studies questioned those findings by showing either neutral or positive associations between adiponectin levels and stroke severity, mortality, and outcome [30]. Beside depending on the amount and distribution of body fat, adiponectin levels seem to differ in patients based on the stroke type with atherothrombotic brain infarctions showing higher values than those with cardioembolic origin [31]. Lastly, a large study by Tu et al. conducted on 4274 patients presenting with acute ischemic stroke and showed that elevated adiponectin values were positively correlated with increased risk of major adverse cardiovascular and cerebrovascular events (MACCEs) and mortality, furthermore, this prognostic role of adiponectin was more pronounced among women and people with increased NT-pro BNP values [32]. Such conflicting results between experimental and clinical evidence have been reported also in other subpopulations of patients such as those at risk of developing type 2 diabetes mellitus, bringing to light what can be defined as “adiponectin paradox” [33]. Further studies are warranted to better understand the molecular and biological mechanisms underlying the role of adiponectin as a biomarker and as a therapeutic target in people at high cardio- and cerebrovascular risk.

### Leptin

Leptin is a 16 kDa peptide first identified in 1994 as a product of the obese (ob) gene after characterizing genetically obese (ob/ob) mice. Serum leptin levels are positively associated with body fat mass, while decreasing during fasting. In addition to its effects in regulating metabolic functions and its possible role in the treatment of obesity leptin plays a role in inflammation and in the immune response by promoting the expression of IL-1Ra and inducing the production of TNF-α and IL-6 [34]. Based on these data, several studies have been conducted on mouse models to clarify the role of leptin in the pathogenesis of stroke, and on neuromotor or behavioral outcomes and mortality. Leptin administered after stroke in mouse models improves outcomes, neuronogenesis and angiogenesis of the perilesional brain tissue [35]. Delayed administration, while maintaining its effect on neuronogenesis and angiogenesis, did not improved outcomes. This result was explained at least in part by the antiapoptotic effects of such adipokine [36]. Further studies investigated this neuroprotective and antiapoptotic effect: the administration of leptin reduced oxidative stress by reducing malondialdehyde and nitric oxide levels, and by increasing the levels of superoxide dismutase [37]; it exerted an antiapoptotic effect by inducing the upregulation of Bcl-2, the downregulation of caspase-3 [37] and activating the nuclear translocation of NF kappa-B/c-Rel through Bcl-xL; promoting metabolism through the PI3k/Akt pathway and modulating mitochondrial function through JAK2/STAT3/PGC-1. Despite the promising role of leptin in improving outcomes following experimental ischemic stroke, to date investigations on its potential therapeutic role in patients are still missing.

Regarding the role of leptin as a risk biomarker of stroke, a first meta-analysis carried out in 2014 positively correlated serum leptin levels with the risk of stroke [38] but subsequent meta-analysis questioned this result by showing no correlation with cardio- or cerebrovascular risk [39]. Few works are currently available regarding the correlation of leptin values ​​with outcomes following ischemic stroke. Low leptin levels upon admission for stroke were negatively correlated with 3-months poor outcome and mortality in patients with type 2 diabetes [40]. Instead, elevated leptin values ​​are correlated with the development of post-stroke depression and poor functional and cognitive outcomes [41]. Higher leptin/adiponectin ratio are correlated with better neurological outcomes in patients with stroke [42].

### Resistin

Resisitin is a 12.5 kDa protein first described in 2001 and produced mainly by adipocytes and white blood cells. Resistin forms oligomers of 660 kDa and trimers of 45 kDa which have a greater biological activity in enhancing the production of pro-inflammatory cytokines IL-1β IL-6, IL-8 and TNFα. Resistin also induces the expression of VCAM-1 and MCP-1 in mouse endothelial cell suggesting a role in atherogenesis. Despite its role in inflammation, resistin appears to exert a neuroprotective effect in murine stroke models [43]. In a model of brain ischemia/reperfusion damage was found that resistin administration prior to the stroke induction, reduced infarct size and improved outcome, reducing cleaved levels of PARP-1 used as a marker of cellular apoptosis [43]. These neuroprotective proprieties were partially reversed after the administration of a PI3K inhibitor, indicating that the PI3K/Akt pathway could be implicated in the antiapoptotic role of resistin [43]. These results were confirmed by a subsequent study which found that the administration of resistin within one hour of induction of ischemia decreased the levels of caspase-3 and caspase 8 and the oxidative stress of the infarcted brain tissue. A later study by the same researchers investigated how intra-cerebroventricular injection of resistin could act on inflammation markers and found that it reduced the expression of pro-inflammatory HSP-70 gene, also increasing the expression of the anti-inflammatory cytokines IL-10 and TGF-β1 [44].

Several studies aimed to investigate the incidence of stroke in patients with high serum resistin values. The Hisayama study showed that high serum resistin levels are associated with the risk of ischemic stroke in peoples with diabetes and hypertension [45]. The PRIME study found that resistin provided an additive value together with the classic risk factors in predicting ischemic stroke in a large cohort of 9771 patients [46]. It was found that the − 420 G/G polymorphism in the promoter region of the resistin gene is associated with higher circulating levels of resistin and an increased risk of developing type II diabetes mellitus. Subsequent studies have shown that − 420 C > G polymorphism may play a role in predicting stroke in diabetic patients and is associated with increased severity of stroke upon admission and increased in-hospital mortality [47]. Elevated serum values of resistin may be associated with increased risk of 5-years mortality and disability. A recent study conducted on 106 patients showed that high resistin levels are associated with worse functional outcome at discharge [48].

Such a discrepancy between experimental and clinical results may be explained by the role of resistin in atherosclerosis onset and plaque development [49], one of the major causes of ischemic stroke in humans which is usually not represented in experimental models of ischemic stroke. Yet, despite its potential role currently are no clinical trials investigated the use of this peptide in patients with stroke.

## Adhesion molecules

**Fig. 2:**
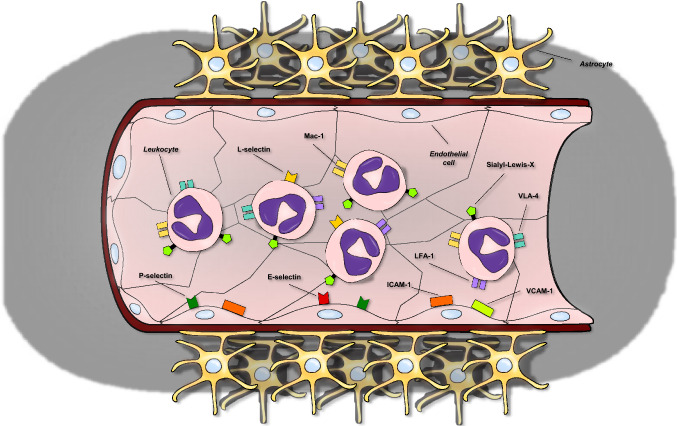
Adhesion molecules as biomarkers in patients with stroke. Several adhesion molecules expressed by different cell types interact to regulate inflammatory cell extravasation towards the infarcted area after the ischemic stroke. *LFA-1* lymphocyte function-associated antigen 1, *ICAM-1* intercellular adhesion molecule 1, *Mac-1* macrophage-1 antigen, *VCAM-1* vascular cell adhesion molecule-1, *VLA, 4* very late antigen-4

Adhesion molecules mainly expressed by endothelial cells at the blood–brain barrier and on leukocytes facilitates their invasion of the ischemic area thereby promoting post-stroke inflammation and fueling a vicious circle leading to even more leukocyte extravasation and parenchymal damage. Classified based on their structure, location or function, major types of adhesion molecules include integrins, cadherins, selectins and immunoglobulin-related adhesion molecules (Fig. 2). Integrins are transmembrane heterodimeric receptors with role in cell–cell and cell-extracellular matrix adhesion. Being receptors further to adhesion molecules, once activated they mediates cellular signaling. Cadherins need calcium on their extracellular domain to be active and from adherent junctions, epithelial-, neural- and placental-cadherin have been described. Selectins hold a single-chain transmembrane structure and share properties to C-type lectins being able to bind sugar moieties. The immunoglobulin (Ig)-related cellular adhesion molecules are the only ones that do not need calcium for their activity. They are characterized by Ig-like domains in their heir extracellular domains and can bind to integrins or to other Ig-related CAMs.

### Intercellular adhesion molecule 1 and vascular cell adhesion molecule 1

Intercellular adhesion molecule 1 (ICAM-1), also known as cluster of differentiation (CD)54 and vascular cell adhesion molecule 1 (VCAM-1) are part of the immunoglobulin superfamily and are among the most studied molecules in the pathogenesis of stroke-related damages. Such molecules are typically found on the surface of endothelial cells, ICAM-1 binds to the integrins LFA-1 (also known as CD11a/CD18) and Mac-1, whereas VCAM-1 is the ligand for VLA4 (also known as integrin α4β1) on the surface of leukocytes. These molecules favor the transmigration of leukocytes through the endothelium of the blood vessels into tissues, including the brain parenchyma. The local expression of ICAM-1 is higher during infectious diseases, traumatic events, or ischemia involving the central nervous system. ICAM-1 plays a direct role in the pathophysiology of ischemic stroke, infarct sizes are smaller in experimental models lacking ICAM-1 or treated with antibodies against it [50]. Of interest, the administration of ICAM-1 antibodies showed a reduction of the ischemic area only animal models of stroke with transient ischemia [51], while no effect was found in permanent ischemia models [52], highlighting the important role of leukocyte infiltration during ischemia/reperfusion injury. Results for a possible role of VCAM are not that straight. Anti VCAM treatment did not show to be beneficial in an experimental model of stroke [53], whereas targeting its ligand VLA-4 with specific antibodies showed mixed results [53, 54]. In particular, the early administration of the antibody against VLA-4 showed the most beneficial results, indicating that the time of administration is probably a critical aspect of such approach.

In humans, elevated levels of VCAM-1 and ICAM-1 are found in the blood and in the infarcted areas of stroke patients [55]. The rise of pro-inflammatory cytokine levels that follows the ischemic injury is thought to increases ICAM-1 and VCAM-1 expression on the endothelial cells of the BBB contributing to the neuroinflammation [56]. Higher levels of soluble ICAM-1 are found in the blood samples of patients with acute stroke that develop neurological deterioration [57] and in the subject that are more likely to die [58]. In addition, a recent meta-analysis showed that the rs5498 polymorphism of ICAM-1 is associated with a higher risk of ischemic stroke in Caucasian subjects [59]. However, other studies found no correlation between soluble ICAM-1 with prognosis of stroke patients [60]. Also, VCAM-1 levels at the admission can predict the outcome of stroke patients but are not associated with the infarct size or disability burden [61]. Of much interest, ICAM has been the target of a randomized clinical trial in which patients with ischemic stroke received an anti-ICAM-1 antibody, enlimomab (Table 1) [62]. While failing to show a beneficial effect on stroke outcome, the RCT even show potential harmful effects of enlimomab (mostly infections and fever) causing an excess of mortality in this group of the study [62].

**Table 1:** Major clinical trials targeting adhesion molecules in patients with stroke

Author	Year	Target	Patients	Major findings/Information
Enlimomab Acute Stroke Trial Investigators [62]	2001	ICAM-1	625 patients with ischemic stroke	Enlimomab (anti-ICAM-1 antibody) did not show efficacy and proved harmful
Krams et al. [78]	2003	CD11b	966 patients treated with neutrophil inhibitory factor (UK-279,276) within 6 h from symptoms	RCT terminated early for futility. No difference in adverse effects among groups
Elkins et al. [79]	2017	Leukocyte adhesion molecule α4 integrin (VLA-4)	161 patients with acute ischemic stroke	Natalizumab did not improve the infarct size. Signals toward improved functional and cognitive test
Elkind et al. [80]	2020	Leukocyte adhesion molecule α4 integrin (VLA-4)	277 patients with ischemic stroke	Natalizumab at different dosages reduced the possibility of having an excellent outcome. No difference in adverse events

*CD* cluster of differentiation, *ICAM-1* intercellular adhesion molecule 1, *VLA, 4* very late antigen-4

### Selectins

E-selectin is the endothelial selectin. Not constitutively expressed, E-selectin is synthetized under inflammatory conditions. In preclinical stroke studies, mice missing E-selectin showed a reduced area of brain infarction and less neurological damages [63]. Lacking E-selectin was associated with less inflammation and apoptosis after stroke. Accordingly, the use of antibodies against E-selectin ameliorates the prognosis in experimental models through a reduction of neutrophil infiltration of the infarcted area [64]. P-selectin is found on the surface of platelets and endothelial cells. A higher P-selectin expression is associated with blood–brain barrier alterations. Accordingly, P-selectin knockdown mice show a reduced infarcted area, less neutrophil influx in the ischemic area, and a higher tendency of blood reflow after brain ischemia [65]. The use of monoclonal antibodies targeting P-selectin in in animal models of stroke ameliorate of the infarcted area even if the drug was administered after the ischemic event [65]. Of much interest, a humanized antibody against E- and P-selectin when administered in the first hour after an ischemic stroke ameliorated its prognosis in non-human primates, treated animals showed a lower infarcted area, a better neurological score, and a reduced infiltration of polymorphonucleate cells in the ischemic brain [66]. L-selectin is typically found on the surface of leukocytes and plays a crucial role in the leukocyte transmigration through the membrane [67]. The potential role of L-selectin in stroke is not yet clear. Evidence is lacking concerning the role of this molecule in stroke. In preclinical animal models of stroke, the administration of anti-L-selectin antibodies together with tissue plasminogen activator can reduce infarcted area and favor the restoration of blood flow [68].

Similarly, also the role of E-selectin as biomarker of stroke in patients is not clear. When some studies report an increase of circulating E-selectin in the early phases reducing after three to six months, other investigations reported no difference between stroke patients and healthy subjects [69]. Yet, plasma levels of E-selectin did show ability to predict the stroke outcome and specific E-selectin polymorphisms are associated with a higher risk of ischemic stroke [70]. P-selectin expression rises in the infarcted areas of the stroke patients’ brain and higher levels of circulating P-selectin is associated with a higher tendency for stroke, possibly related to an underlying thrombotic state in patients aged more than 65 years old [71]. However, the possibility that P-selectin might correlate with activated platelets showed mixed results in stroke patients. Gene polymorphism analyses showed an association L-selectin P213S polymorphism with stroke [72], while no association was reported for P-selectin gene polymorphism [73].

### Integrins

In the field of ischemic brain disease, lymphocyte function-associated antigen 1 (LFA-1) and macrophage-1 antigen (Mac-1) are the most studied integrins to date as they mediate leukocyte adhesion to the endothelium. LFA-1 connects to ICAM-1 and is composed of CD11a (α-subunit) and CD18 (β-subunit). Similarly, Mac-1 (CD11b/CD18) is expressed on the surface of leukocyte membrane and binds to the same ligand. In experimental models of stroke, Mac-1 or LFA-1 deficiency associates with reduced parenchymal damage [74]. Similarly, CD18 knockout mice have a reduced infarct area after transient cerebral ischemia [75]. Furthermore, targeting CD11b and/or CD18 with specific inhibiting antibodies improves the ischemic damage in animal models of stroke, especially when transient ischemia occurs [76]. Although reports are not always consistent.

Clinically, CD11a and/or CD18 are up-regulated in patients during the acute phases of ischemic stroke. Plasma levels of Mac-1 at the time of admission are associated with the severity of the stroke [77]. Such observational study together with the promising experimental findings led to the design of a RCT using UK-279,276 (neutrophil inhibitory factor), a recombinant glycoprotein with selective binding to the CD11b in stroke patients. Despite being overall safe, the drugs failed to meet the expected outcome (improvement of neurologic functions based on the Swedish Stroke Scale) and the trial was terminated early for futility [78].

## Conclusions

The inflammatory environment found in the nervous system because of stroke is nowadays recognized as one of the leading determinants of parenchymal damages. In this context, inflammatory mediators including cytokines, adipokines and adhesion molecules may represent possible targets for the development of specific treatments. Such approaches—despite holding promising results in the preclinical setting—constantly failed the bench-to-bed transition either due to futility or even harmfulness. Indeed, post-stroke inflammation plays a dual role in the pathophysiology of ischemic stroke. Beside participating in the parenchymal damage immediately after the ischemic insult, inflammatory molecules and cells are thought to be important mediators of cerebral recovery and neuronal plasticity in the long term. Furthermore, the deleterious effects of inflammation during the first phase after ischemic stroke may differ from patient to patient based on disease- (e.g. reperfusion, type of stroke) as well as on subject-related (e.g. comorbidities, age) characteristics. A less broad modulation of inflammation, based on a patient-specific approach still hold potential to reduce the burden of such major killer. Such ambitious goal needs a detailed knowledge of the complex interplay between inflammatory mediators at the different stage of the disease and in different patients therefore asking for further research in this promising field.

## Data Availability

This is a narrative review, without data from scietific experiments.
